# Tautomerization, acidity, basicity, and stability of cyanoform: a computational study

**DOI:** 10.1186/s13065-016-0166-z

**Published:** 2016-04-11

**Authors:** Shaaban A. Elroby

**Affiliations:** Chemistry Department, Faculty of Science, King Abdulaziz University, P.O. Box 80203, Jeddah, 21589 Saudi Arabia; Chemistry Department, Faculty of Science, Beni-Suef University, Beni-Suef, 62511 Egypt

**Keywords:** Cyanoform, Tautomerization, Water-assisted proton transfer, B3LYP, MP2, PCM, Raman spectra

## Abstract

**Background:**

Cyanoform is long known as one of the strongest acid. Cyanoform is only stable below −40 °C. The issue of the stability and tautomeric equilibria of cyanoform (CF) are investigated at the DFT and MP2 levels of theory. The present work presents a detailed study of structural tautomer interconversion in three different media, namely, in the gas phase, in a solvent continuum, and in a microhydrated environment where the first solvation layer is described explicitly by one or two water molecule. In all cases, the transition state has been localized and identified. Proton affinities, deprotonation energies and the Raman spectra are reported analyzed and discussed.

**Results:**

The **1** tautomer of cyanoform is shown to be more stable than **2** form by only 1.8 and 14.1 kcal/mol in the gas phase using B3LYP/6-311 ++G** and MP2/6-311 ++G** level of theory, respectively. This energy difference is reduced to 0.7 and 13.4 kcal/mol in water as a solvent using CPCM model using B3LYP/6-311 ++G** and MP2/6-311 ++G** level of theory, respectively. The potential energy barrier for this proton transfer process in the gas phase is 77.5 kcal/mol at MP2/6-311 ++G** level of theory. NBO analysis, analysis of the electrostatic potential (ESP) of the charge distribution, donor–acceptor interactions and charge transfer interactions in **1** and **2** are performed and discussed.

**Conclusions:**

Gross solvent continuum effects have but negligible effect on this barrier. Inclusion of one and two water molecules to describe explicitly the first solvation layer, within the supermolecule model, lowers the barrier considerably (29.0 and 7.6 kcal/mol, respectively). Natural bond orbital (NBO) analysis indicated that the stability of the cyanoform arising from charge delocalization. A very good agreement between experimental and theoretical data has been found at MP2/6-311 ++G** for the energies. On other hand, B3LYP/6-311 ++G** level of theory has good agreement with experimental spectra for CF compound.

**Electronic supplementary material:**

The online version of this article (doi:10.1186/s13065-016-0166-z) contains supplementary material, which is available to authorized users.

## Background

Tricyanomethane or cyanoform is long known as one of the strongest acid with pKa = −5.1 in water and 5.1 in acetonitrile [1], however, its relative stability have been and still is a controversial subject. The molecule has previously only been identified by microwave spectroscopy in the gas phase at very low pressures [2–4].

**Scheme 1:**
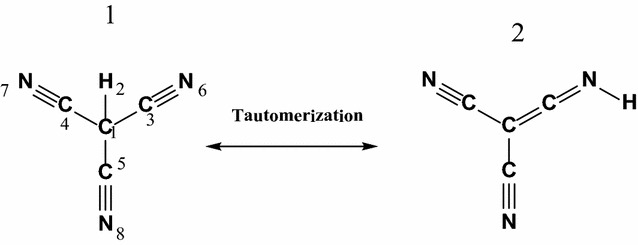
Tautomers form of cyanoform **1** and **2**

Since the first attempt of its synthesis and isolation in 1896, numerous attempts to isolate cyanoform have been reported, but none of them were successful. Dunitz et al. reviewed these attempts and reinvestigated most of them [5]. The tautomeric dicyanoketenimine (**2)**, tricyanomethanide (**1**), scheme 1) was suggested to play a role in the stability and high acidity of **1**. Structure **1** is only stable below −40 °C [6]. Its extreme high acidity was interpreted on the basis that its structure has three cyano groups attached to CH group. The deprotonation of hydrogen from center carbon is very easily, making it a strong acid and demonstrating a fundamental rule of carbon acids. The rule describes how electron-loving groups attached to a central hydrogen-toting carbon pull on that carbon’s electrons.

The stability and structure of **1** in the gas phase were investigated by quantum chemical calculations [7–13]. Results of these computational studies revealed that 1 is more stable than **2** by about 7–10 kcal/mole in the gas phase. In the present work, the issue of the stability and tautomeric equilibria of **1** are revisited. Computations at high level of theory and in the gas as well as in solution are performed. Water-assisted proton transfer is investigated for the first time where transition states, a barrier energies and thermodynamic parameters are computed. The ground state geometries, proton affinities, deprotonation energies and

the Raman spectra are reported. NBO analysis of the charge distribution, donor–acceptor interactions and charge transfer interactions in 1 and 2 are performed and discussed.

## Computational methods

All quantum chemical calculations are carried out using the Gaussian 09 [14] suite of programs. Full geometry optimizations for each and every species studied have been carried out using two DFT functionals namely, the B3LYP [15–17], and MP2 [18–20] methods using the 6-311 ++G** basis set. The frequency calculations carried out confirm that all the optimized structures correspond to true minima as no negative vibration frequency was observed. Number of imaginary frequencies are zero for minima and one for transition states. Zero point energy (ZPE) was enclosed in all energetic data.

Among all DFT methods, B3LYP often gives geometries and vibration frequencies, which are closest to those obtained from the MP2 method. Natural bond orbital (NBO) population analysis on optimized structures is accomplished at the B3LYP/6-311 ++G** level [21]. NBO calculations were performed using NBO 5.0 program as implemented in the gaussian 09 W package. The effect of solvent (water) is taken in consider using the self-consistent reaction field polarisable continuum model (SCRF/PCM) and SMD models [22–24]. Results were visualized using chemcraft program [25].

## Results and discussion

**Fig. 1:**
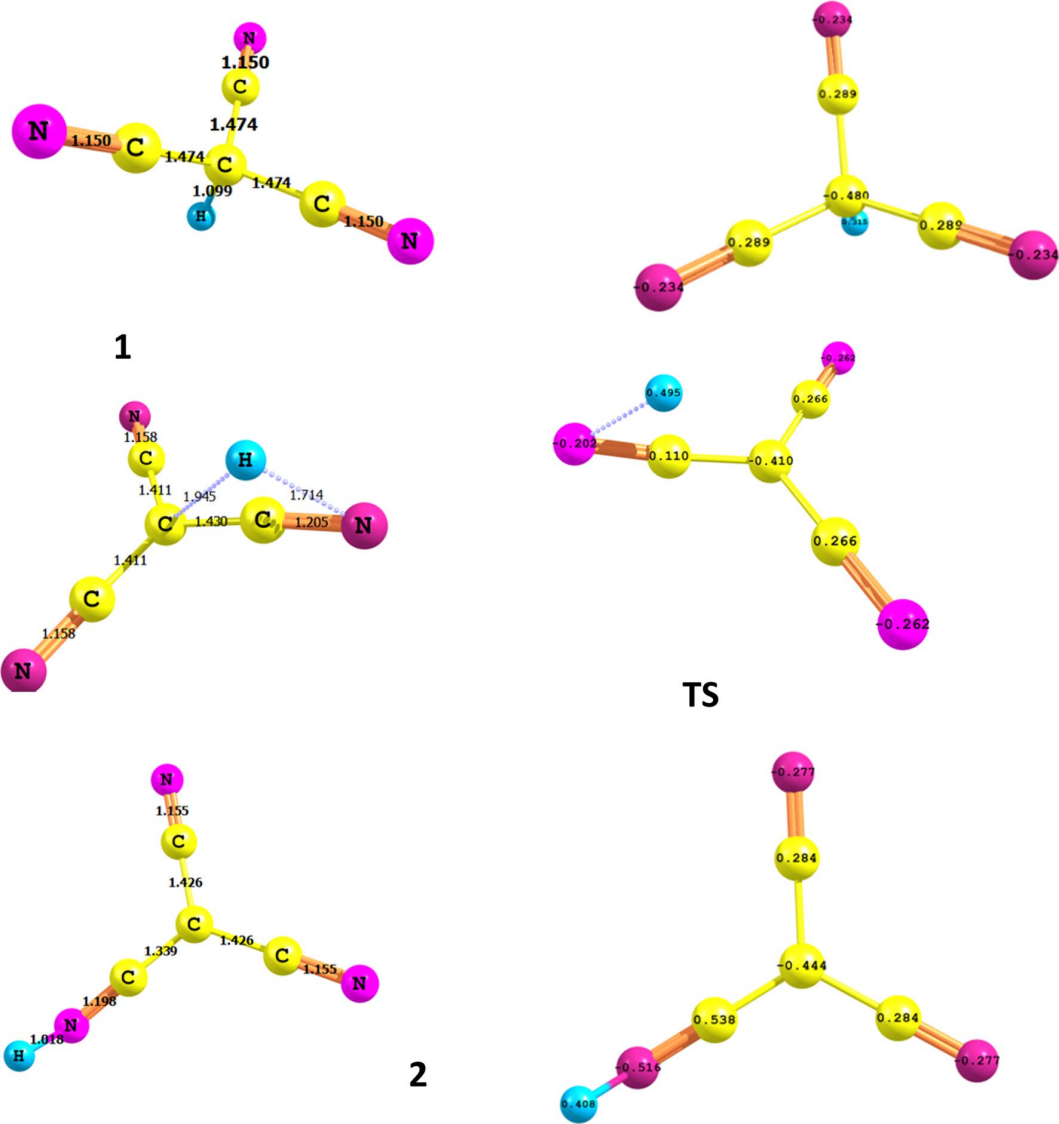
Optimized structures of CF-CH, TS and CF-NH structures obtained at the B3LYP/6-311 ++G** level. Bond length is in Angstrom, charge distribution is natural charge

Figure 1 displays the fully optimized structure of **1**, TS, and **2**. These structures represent the global minima on the respective potential energy surfaces computed at two different levels of theory, namely, B3LYP and MP2/6-311 ++G**. The two theoretical models gave very comparable geometries. **1** is highly symmetric tetrahedral structure with all C–C–C 110.9^o^ and the C–C-H angle 108.0°. That is the central carbon atom assumes a typical sp^3^ hybridization scheme. Tautomer **2**, on the other hand, is planar having the central carbon atom assuming an sp^2^ hybridization scheme with C–C–C angles of 120^o^. The hydrogen atom in **2** form is tilted out of the molecular plane by an angle of 53^o^. The two tautomers (**1** and **2**) show also some minor structure variations reflected in the shortening of the C–C and slight elongation of the C-N bond lengths upon going from **1** to **2**. Figure 1 displays also the net charges on each atom of **1** and **2**. It can be easily noticed that the C-N–H moiety is highly polarized with a considerable charge (0.538, −0.516 and 0.408e, on the C, N and H, respectively) separation. This charge separation is much greater than that observed for the **1** tautomer (0.289 and −0.480 on the C and N, respectively).

Due to the **1** **→** **2** intramolecular-proton transfer, a number of structural parameters of the **1** form have changed. Going from the **1** to the **2** tautomer, the C–C bonds length decreases from 1.475 to 1.430 and 1.342 Å, whereas the C–N bond length enlarges from 1.175 to 1.178 Å. In the optimized geometry of the TS, breaking of the C–H1 bond together with the formation of N8–H1 bond is clear. In **1** tautomer, The C1–H1 and C–C distances vary from 1.098 and 1.474 Å for the **1** tautomer to 1.862 and 1.426 Å for the TS, respectively. The N1–H1 is 1.539Å in TS. This distance is 1.019 Å for the 2 tautomer. The analysis of the normal modes of TS imaginary frequencies (−1588.00) revealed the displacements of N6–H2 and C1–H2 bond lengths of **1**.

### Tautomerization 1⇄2

Proton transfer reactions are very important in chemistry and biology as it underlie several technological and biological processes.

Some investigations [6] have suggested that the tautomeric form **2** may exist and underlies the strong acidity of cyanoform. In the present section, the possibility of 1, 3 proton transfer in **1** will be explored.

Table 1 compares the relative energies of the two tautomers **1** and **2** computed at two different level of theory. The two methods indicated that the **1** form is more stable than **2** form by 14.1 and, 1.8 kcal/mol, at the MP2/6-311 ++G** and B3LYP/6-311 ++G** levels of theory in the gas phase, respectively. It seems that B3LYP is not able to account for some stabilizing interactions in **1** in particular electron correlations which is well accounted by MP2 calculations.

**Table 1:** Total and relative energies for the studied species using two methods (B3LYP and MP2) at 6-311 ++G** basis set in the gas phase and in the solution

Structure	Gas phase						Solvent		
	MP2			B3LYP				MP2	B3LYP
	E_t_/au		kcal/mol	E_t/au_		kcal/mol		kcal/mol	
1	−316.40004		0.0	−317.27785		0.000		0.0	0.0
2	−316.37761	E_re_	14.1	−317.27506	E_re_	1.8	E_re_	13.4	0.7
TS	−316.28143	E_a_	77.5	−316.79868	E_a_	68.7	E_a_	74.4	68.4
CF^−^	−315.91668	DP	303.3	−317.16841	DP	300.7	DP	272.7	262.6
(CFH)^+^	−316.70615	PA(H)	−46.8	−317.54566	PA(H)	−168.1	PA(H)	−230.1	−231.4

*E*_*t*_ electronic energy, *E*_*re*_ relative energy between two tautomeric forms, *E*_*a*_ barrier energy, *DP* deprotonation energy, *PA* protonation energy

Table 1 compiles also relative energies in water as a solvent computed using the solvent continuum model CPCM, where the **1** tautomer is found to be the more stable. Solvent dielectric constant seems to have marked effect on the stability of **1**. This is in agreement with a previous experimental study [6].

The lower relative stability of the **2** tautomer may be due to the close proximity of the lone pairs of electrons on the N8 atom and the adjacent triple bond in **2** forms, in **2** form H–N–C angle is bent. On the other hand, the lone pairs of electrons on all N atoms in **1** tautomer are projected in opposite directions collinear with triple bonds. This will minimize the repulsive force in the **1** tautomer as compared to that in the **2**.

The 1, 3 proton transfer process takes place via the transfer of the H atom from the central carbon atom to N8. We have been able to localize and identify the transition state (TS) for this process, which is displayed in Fig. 1. Some selected structural parameters of the TS are collected together with the corresponding values for **1** and **2** tautomers for comparison (Additional file 1: Tables 1S and 2S and Figure 1S.

The barrier energy computed for this tautomerization reaction is 68.7 and 74.4 kcal/mol at B3LYP/6-311 ++G** and MP2/6-311 ++G** level of theory in the gas phase, respectively.

**Fig. 2:**
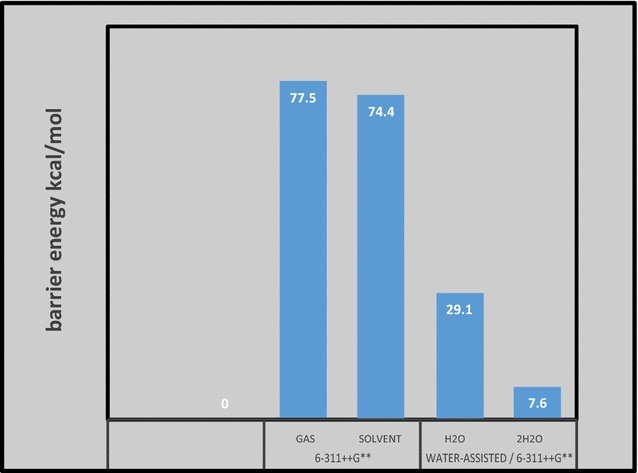
The barriers energy for the proton-transfer process of **1** assisted by one and two water molecule, with and without PCM–Water. Energies are in kcal/mol at the MP2 method at basis set 6-311 ++G**

In the present work, results generated by DFT and MP2 methods at 6-311 ++G** basis set, barrier energy (E_a_) of the **1** and **2** tautomerism in aqueous solution is 68.4 and 77.5 kcal/mol, respectively. This high energy barrier seems to indicate that this reaction is not feasible at room temperature. Solvent dielectric continuum seems to have but little effect on this barrier; in fact, it reduced it by less than 1 % (see Fig. 2).

Considering the equilibrium between the **1** and **2** tautomers, the value of the tautomeric equilibrium constant (K) is calculated by using

1 $$\text{K}={{\text{e}}^{-\Delta \text{G}/\text{RT}}}$$

where ΔG, R and T are the Gibbs free energy difference between the two tautomers, the gas constant and temperature, respectively.

The Gibbs free energy difference between the tautomers is in favor of the **1** tautomer by 13.0 kcal/mol using MP2/6-311 ++G** level of theory. By using the Eq. (1), K equal about 3.14 × 10^−10^.

**Scheme 2:**
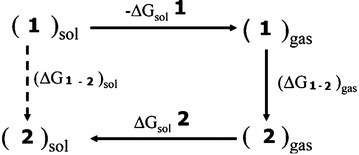
An energy cycle used to calculate relative free energies of tautomers in water solution

To calculate the relative free energies of two tautomers, **1** and **2**, in water solution, (ΔG_**1**−**2**_)_sol_ we use a simple energy cycle of scheme 2:

$$\left( \Delta \text{G}\mathbf{1}-\mathbf{2} \right)\text{sol}=-\Delta \text{Gsol1}+\left( \Delta \text{G}\mathbf{1}-\mathbf{2} \right)\text{gas}+\Delta \text{Gsol}\mathbf{2}$$

where (ΔG_**1−2**_)_gas_ is the free energy difference between **1** and **2** in the gas phase and ΔG_sol_**1** and ΔG_sol_**2** are the free energies of solvation of **1** and **2**, respectively.

The calculated relative energy and relative free energy of two tautomers in the water solution are presented in Table 2. The 1 form is the most stable tautomer than 2 by relative energy and free energy. The relative free energy between 1 and 2 tautomers are 26.8 and 26.4 kcal/mol using the SMD and CPCM models, respectively. The 2 tautomer is less stable than 1 by 14.6 and 14.1 kcal/mol using the SMD and CPCM solvation models, respectively.

**Table 2:** The relative energies and relative free energies for the two tautomer’s using SMD and CPCM models at MP2/6-311 ++G** level of theory in water solution

Structure	SMD		CPCM	
	E_re_	ΔG	E_re_	ΔG
**1**	0.0	0	0.0	0.0
**2**	14.6	*26.8*	14.1	26.4

The unit of energies is kcal/mol

### Water-assisted proton transfer

**Fig. 3:**
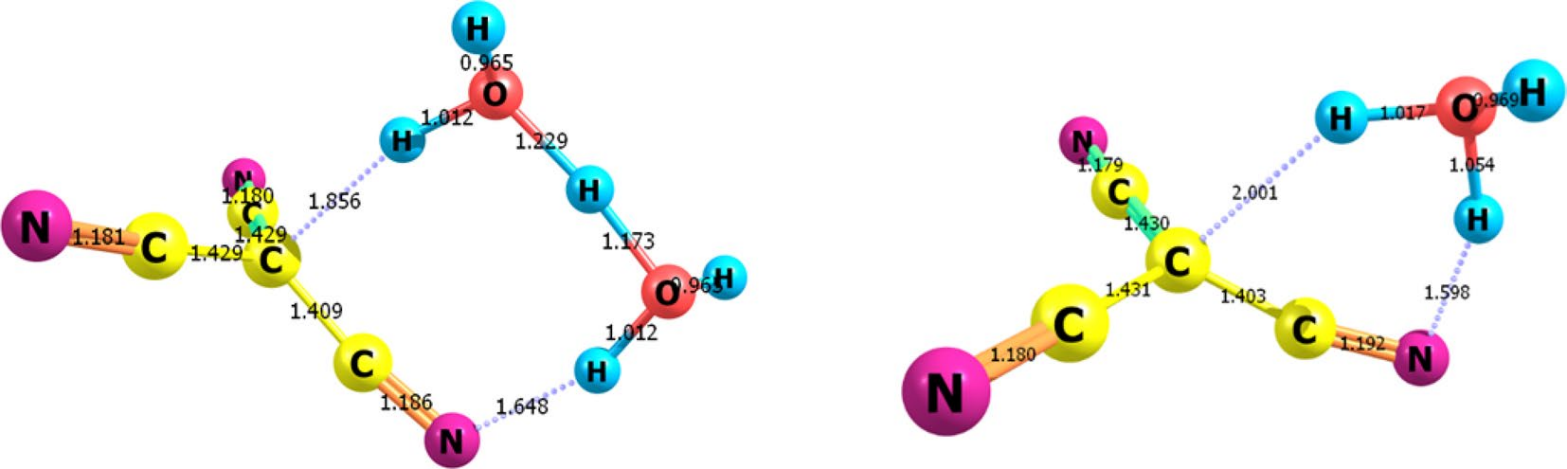
Optimized structures, of two (*left*) and one (*right*) water-assisted transition states for the tautomerization of cyanoform computed at MP2/6-311 ++G** level of theory

The structure computed in the gas-phase for TS (Fig. 3) reveals the formation of a triangular 4-membered ring. The high energy and relative instability of this TS is associated with the large strain in this triangular ring. In solution, however, one way to relief this strain is to incorporate one or more water molecules in the formation of the transition state. We have examined the possibility of water-assisted proton transfer for the studied tautomerization reaction using MP2/6-311 ++G** level of theory. We have incorporate one and two water molecules. The TS’s so obtained are displayed in Fig. 3 and the corresponding energy quantities are compiled in Table 1. The presence of one water molecule in the structure of the transition state considerably relief the ring strain and stabilize it considerably to lie at only 29.6 kcal/mol above the **1** form as shown in Fig. 2. The incorporation of two water molecules, stabilize TS reflecting the stability associated with 8-membered ring formed. The barrier energy with two water molecules is about 7.6 kcal/mol. The energy profile presented in Fig. 2 shows that the most important difference between the prototropic tautomerism of dihydrated species and the isolated compound is associated with the activation barriers, which become almost ten times or even less than ten times of those obtained for the isolated compound; this is a well-known phenomenon [26–32]. Thermodynamics of tautomerization of **1,** Table 3 compiles the computed thermodynamic parameters at room temperature and at −40 °C.; at this temperature **1** is known to be stable [6]. Entropies, and enthalpies increase on going from 260 to 300 K, this may be attributed to the fact that intensities of molecular vibration increase with increasing temperature. The enthalpy change (∆H) and the entropy change (∆S) for the reaction are also obtained and listed in Table 3. For the tautomerization of cyanoform **1** to **2**, ∆S is negative while the ∆H is positive at both 260 and 300 K. That is, the proton transfer in cyanoform is an endothermic process. The change in Gibbs free energy (∆G) at two different temperatures was also obtained, and is shown in Table 3. ∆G at 260 K is positive, which demonstrates that the formation process of the CF- NH is not spontaneous.

**Table 3:** Thermal energy parameters for the studied species using B3LYP/6-311 ++G** level of theory in solution at 260 and 300 K

	T = 260 K			T = 300 K		
	H/au	G/au	S/Cal/Mol.K	H/au	G/au	S/Cal/Mol.K
**1**	−317.2288	−317.260935	77.565	−317.22743	−317.265745	80.639
**2**	−317.22652	−317.25843	77.013	−317.22514	−317.263208	80.121

### Protonation and deprotonation

The proton affinity (PA) values help in understanding fragmentation patterns in mass spectroscopy influenced by protonation and other proton transfer reactions, the basicity of molecules and susceptibility toward electrophilic substitution. Knowledge of preferred site of protonation is also of significance for structure elucidation of polyfunctional molecules [33].

For each protonation and deprotonation site, the structure with the lowest energy was identified as the most stable and with respect to this, the relative energies are calculated.

**Fig. 4:**
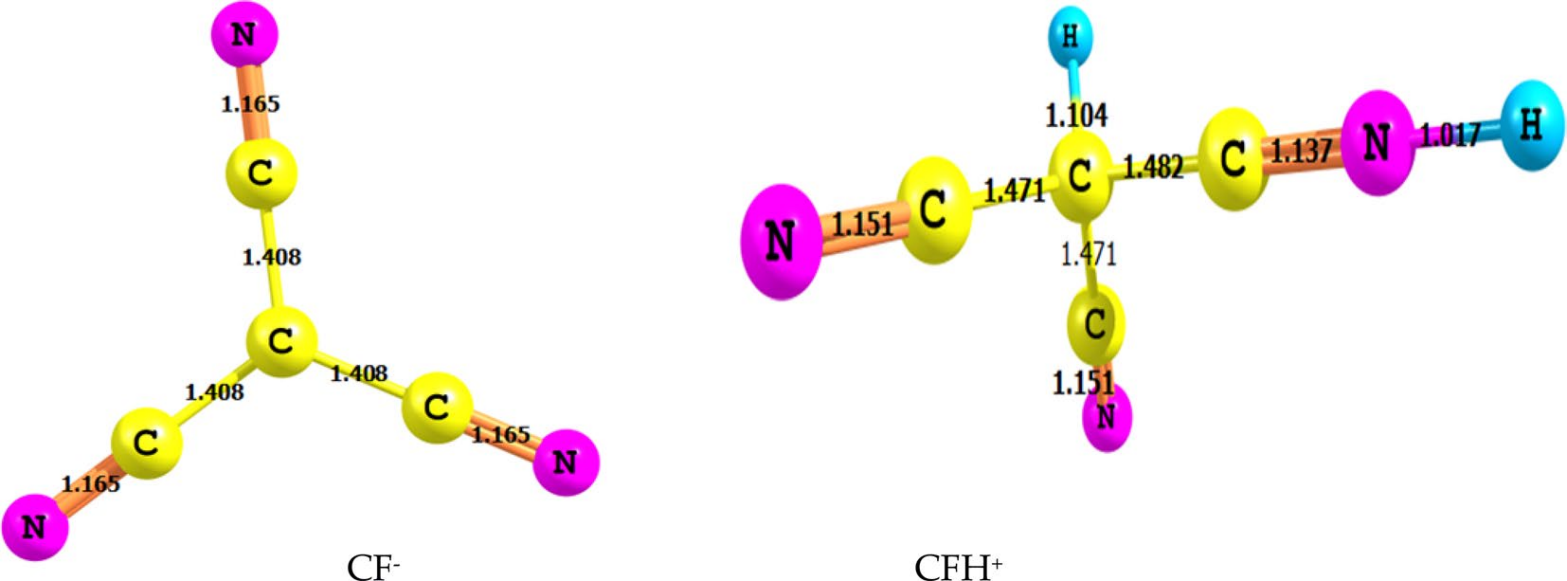
Optimized structures of deprontaed and protonation species of 1 obtained at the B3LYP/6-311 ++G** level of theory. Bond length is in Angstrom

The variation in geometrical parameters on CH-deprotonation and N-protonation at the B3LYP/6-311 ++G** level theory are displayed in Fig. 4. The analysis of variation in geometrical parameters as a result of protonation of the N in **1**, indicates elongation for adjacent C–C bond to protonated N atom along with compression of C–N bond. The protonation energy, ΔE_prot_, was calculated as follows: ΔE_prot_ = E_AH_^+^−E_A_ (where *E*_AH+_ is the energy of cationic acid (protonated form) and *E*_*A*_ is the energy of the neutral form). By the same equation, the deprotonation energy, DP, was calculated using ΔE_DP_ = E_A_^−^—E_A_ (where *E*_A_^−^ is the energy of anion (deprotonated form) and *E*_*A*_ is the energy of the neutral form. The proton affinities for **1** sites at B3LYP/6-311 ++G** in the gas phase are higher than the values evaluated in solution using PCM method while vice versa is observed for the deprotonation (DP) of the C-H bond. Table 1 compiles the deprotonation and protonation energies of the studied species, obtained at the B3LYP/6-311 ++G** and MP2/6-311 ++G** level of theory. The deprotonation energies of the CH bond in the gas phase and in the solution are 303.7 and 272.0 kcal/mol at MP2 method, respectively, i.e. the CH bond is characterized by a strong acidity (1156 kJ/mol) which is sensibly higher than that of NH bonds in formamide (1500 kJ/mol), N-methylformamide (1510 kJ/mol) or N-methylacetamide (1514 kJ/mol) [34]. The reason for this high acidity is probably a strong delocalization of the negative charge over three cyano groups around CH bond.

### Vibration Raman spectrum analysis

**Fig. 5:**
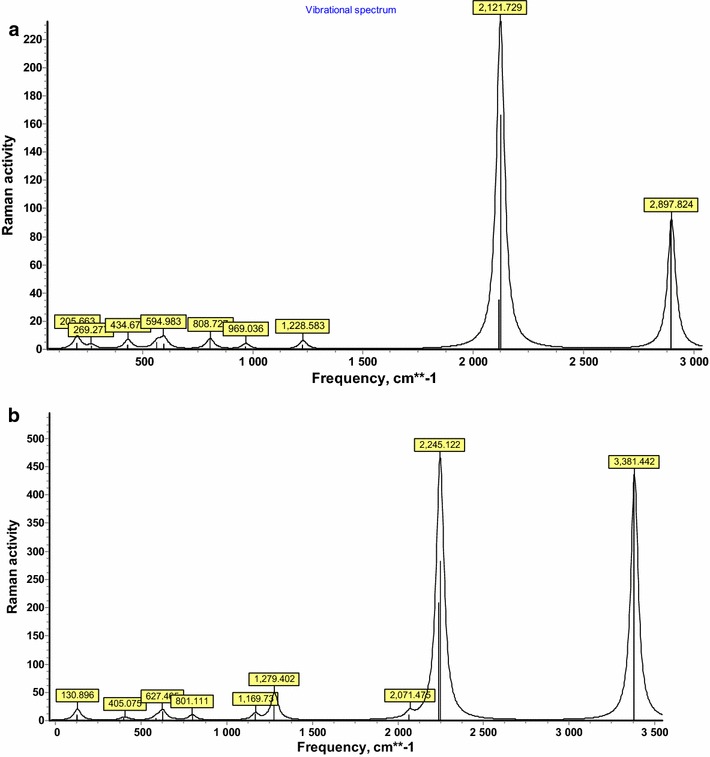
Calculated Raman frequencies (cm^−1^) (**a**) **1** and (**b**) **2** calculated at B3LYP/6-311 ++G** level of theory in the gas phase. Values were scaled by an empirical of 0.96

The experimental [6] and theoretically predicted FT-Raman spectra (intensities) for **1** are represented in Fig. 5 and detailed band information is summarized Table 4. FT-Raman spectrum were calculated by the two methods, DFT B3LYP and MP2 using two basis sets, namely 6-311 ++G** and aug-cc-pVQZ, and the frequency was scaled by 0.96 [35].

**Table 4:** Observed [6] and calculated Raman frequencies (cm^−1^) (scaled by an empirical factor of 0.96) for **1** using B3LYP and MP2 methods at two basis sets 6-311 ++G** and *aug-cc-pVQZ*

B3LYP		MP2		PBE1PBE		
6-311 ++G**	Aug-cc-pVQZ	6-311 ++G**	Aug-cc-pVQZ	6-311G (3df,3dp)	Observed	Assignment
342 (3)	337 (2)	323 (3)	316 (2)	345	347 (45)	∂ *CCN*
549 (5)	551 (5)	544 (4)	541 (5)	556	567 (16)	∂ *CCN*
555 (2)	553 (1)			559	575 (7)	∂ *CCC*
804 (6)	808 (7)	808 (7)	801 (8)	813	835 (24)	*v*_*s*_ *CC*
985 (1)	980 (1)	995 (2)	994 (1)	1002	1022 (7)	*v*_*as*_ *CC*
1238 (3)	1239 (3)	1247 (3)	1239 (2)	1232	1253 (5)	∂ *CCH*
2281 (34)	2284 (31)	2093 (82)	2098 (98)	2310	2259 (7)	*v*_*as*_ *CN*
2288 (160)	2292 (175)	2101 (18)	2105 (18)	2316	2287 (100)	*v*_*s*_ *CN*
2895 (88)	2894 (85)	2960 (85)	2956 (82)	2922	2885 (38)	*v CH*

The Raman spectrum of cyanoform was reported recently by Theresa Soltner et al. [6]. Comparison of the of the theoretically computed frequencies and those observed experimentally shows a very good agreement especially with B3LYP/aug-cc-pVQZ level of theory.

Most intensive band in Raman spectra, obtained experimentally was observed at 2287 cm^−1^ occurred in calculated spectra at 2288, 2292 and 2316 cm^−1^ in B3LYP/6-311 ++G**, B3LYP/aug-cc-pVQZ and PBE1PBE/6-311G(3df, 3dp) [6] level of theory, respectively.

**Fig. 6:**
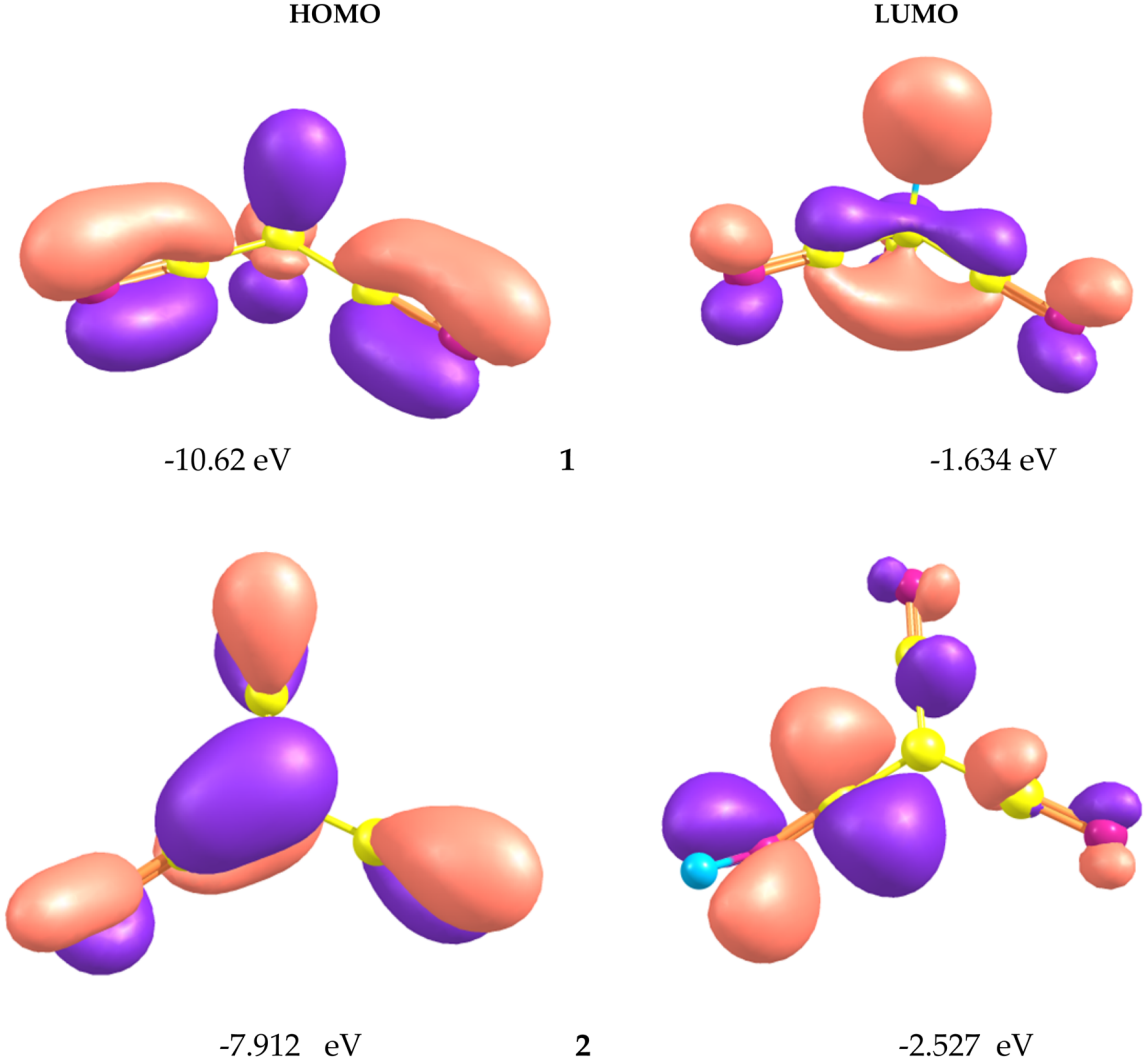
The HOMO and LUMO frontier orbitals of the **1** and **2** tautomers. (The Isovalue = 0.05) using B3LYP/6-311 ++G** level of theory

MP2 simulated spectra were found have less vibrational band deviation and missing one band from the observed spectrum for the studied molecule, as shown in Fig. 6 and Table 4. It is interesting to note that, the C–H asymmetric stretching vibrations is observed experimentally at 2259 cm^−1^ and predicted theoretically at 2098 and 2093 cm^−1^ using the MP2/6-311 ++G** and MP2/aug-cc-pVQZ level of theory, respectively, in weak agreement. DFT functionals show a good prediction spectra of nitriles and their anions [36–40].

It should be noted that the B3LYP at the two basis sets gave good band position evaluation, e.g. band appeared at 2285 cm^−1^ (obs), 2895 cm^−1^ (6-311 ++G**) and 2894 cm^−1^ (aug-cc-pVQZ).

As it can be seen from Table 4, the theoretically calculated values at 2897 and 1228 cm^−1^ showed excellent agreement with the experimental values.

The C–H stretching vibrations is observed experimentally at 2885 cm^−1^ and predicted theoretically at 2895 and 2894 cm^−1^ using the 6-311 ++G** and aug-cc-pVQZ basis sets, respectively, in excellent agreement.

The γ(C-N) stretching is predicted theoretically at 2288 cm^−1^ using 6-311 ++G** basis set in a very good agreement with the experimental observed Raman line at 2287 cm^−1^. No bands for C=C or C=N stretching vibrations are observed in FT-Raman of **1**. The absence of any band in the 1500–1900 range confirms that the stable form for the studied molecule is **1** tautomer. Full assignment of Raman spectrum of **1** tautomer is given in Table 4.

### NBO analysis

NBO analysis has been performed on the molecule at the MP2 and B3LYP/6-311 ++G** level of theory in order to elucidate the intra molecular, hybridization and delocalization of electron density within the studied molecule, which are presented in Table 5.

**Table 5:** Second order perturbation energy (E^(2)^) in NBO basis for **1** using B3LYP and MP2 methods at 6-311 ++G** basis set

Donor	Type	Acceptor	Type	E^(2)^			
				B3LYP/6-311 ++G**		MP2/6-311 ++G**	
				1	2	1	2
*C1–C3*	*σ*	*C4–N7*	π*	3.53	5.26	4.25	6.14
*C1–C3*	*σ*	*C5–N8*	π*	3.53	4.21	2.63	5.31
*C1–C4*	*σ*	*C3–N6*	π*	3.53	20.34	4.25	5.68
*C1–C4*	*σ*	*C4–N7*	π*	5.69	7.37	9.19	9.62
*C1–C4*	*σ*	*C5–N8*	π*	3.53	4.44	4.25	5.68
*C1–C5*	*σ*	*C3–N6*	π*	3.53	4.21	4.25	5.31
*C1–C5*	*σ*	*C4–N7*	π*	3.53	5.26	4.25	3.42
*C1–C5*	*σ*	*C5–N8*	π*	5.69	7.48	9.91	4.27
*C3–N6*	*π*	*C1–C3*	σ*	5.62		2.64	9.09
*C3–N6*	*π*	*C1–H1*	σ*	2.76		3.58	
*C4–N7*	*π*	*C1–C4*	σ*	5.62	6.68	8.60	8.62
*C4–N7*	*π*	*C1–H1*	σ*	2.76		3.85	
*C4–N7*	*π*	*C1–C3*	σ*	2.19	3.57	2.65	4.32
*C5–N8*	*π*	*C1–C5*	σ*	5.62	7.36	8.60	9.09
*C5–N8*	*π*	*C1–H1*	σ*	2.76		3.85	
*C5–N8*	*π*	*C1–C3*	σ*	2.19	3.34	2.64	4.12
*C5–N8*	*π*	*C1–C4*	σ*	2.19	6.46	2.64	5.21
*N6*	*LP*	*C1–C3*	σ*	12.13	11.67	12.72	12.52
*N7*	*LP*	*C1–C4*	σ*	12.13	31.61	12.72	78.33
*N8*	*LP*	*C1–C5*	σ*	12.13	11.67	12.72	12.52

E^(2)^ means energy of hyper conjugative interaction (stabilization energy)

*Non-bonding orbitals

Natural bond orbital (NBO) [41, 42] analysis gives information about interactions in both filled and virtual orbital spaces that could help to have a detailed analysis of intra and intermolecular interactions. The second order Fock matrix was carried out to evaluate the donor–acceptor interactions in the NBO analysis [43].

For each donor NBO (i) and acceptor NBO (j), the stabilization energy associated with i–j delocalization can be estimated as,

$${{\text{E}}^{(2)}}=\text{ }\Delta {{\text{E}}_{\text{ij}}}=\text{qi}=\text{F}{{\left( \text{i},\text{j} \right)}^{2}}/{{\varepsilon }_{\text{i}}}{{\varepsilon }_{\text{j}}}$$

where q_i_ is the donor orbital occupancy, ɛ_i_, ɛ_j_ are diagonal elements (orbital energies) and F(i,j) is the off-diagonal NBO Fock matrix clement. The stabilization of a molecular system arises due to overlapping of orbital between bonding and anti-bonding which sequels in an intramolecular charge transfer (ICT).

In Table 5 the perturbation energies of significant donor–acceptor interactions are comparatively presented for **1** and 2 forms. The larger the E^(2)^ value, the intense is the interaction between electron donors and electron acceptors.

The NBO results show that the specific lone pairs of N atoms with *σ*∗ of the C–C bonds interactions are the most important interactions in **1** and CF_NH, respectively.

In **1**, the interactions initiated by the donor NBOs like σ_C1–C2_, σ_C3–C4_, π_N–C_ and NBOs due to lone pairs of N atoms are giving substantial stabilization to the structures in the both MP2 and B3LYP methods. Above all, the interaction between lone pairs namely, N6, N7 and N8 is giving the most possible stabilization to **1** since it has the most E^(2)^ value around 12.81 and 11.5 kcal/mole in **2**. The other interaction energy in the **1** and **2** is π electron donating from π _(C3–N6)_−π*_(C1–C3)_, π_(C3–N6)_−π*_(C1–H2)_, π_(C4–N7)_−π*_(C1–C4)_, and π _(C5–N8)−_π*_(C1–C5_) resulting stabilization energy of about 5.62, 2.76, 5.69 and 5.89 kcal/mol, respectively. The present study at the two methods (MP2 and B3LYP), shows clearly that the electron density of conjugated triple bond of cyano groups exhibits strong delocalization.

The NBO analysis has revealed that the lone pairs of N atoms and C–C, C–H and C–N bonds interactions give the strongest stabilization to both of the **1** and **2** with an average value of 12.5 kcal/mole.

The 3D-distribution map for the highest-occupied-molecular orbital (HOMO) and the lowest-unoccupied-molecular orbital (LUMO) of the **1** and **2** tautomers are shown in Fig. 6. As seen, the HOMO is mainly localized on the cyano groups; while, the LUMO is mainly localized on the CC bonds.

The energy difference between the HOMO and LUMO frontier orbitals is one of the most important characteristics of molecules, which has a determining role in such cases as electric properties, electronic spectra, and photochemical reactions. The gap energy (HOMO–LUMO) is equal to 9.00 and 5.40 eV for the **1** and **2** tautomers, respectively. The large energy gap for **1** tautomer implies that structure of the cyanoform is more stable.

## Conclusions

A comparative study of two different theoretical methods was performed on the cyanoform to obtain the highest accuracy possible and more reliable structures.

- Despite the B3LYP and MP2 methods affording good results which provide a better picture of the geometry and spectra and energetics, respectively, both in the gas phase and in a water solution (PCM–water).
- At all levels of theory used, the **1** form is predicted to be more stable than its **2** form, both in the gas phase and in solution.
- The potential energy barrier for this proton transfer process in the gas phase is 77.5 kcal/mol using MP2/6-311 ++G** level of theory. Gross solvent continuum effects have negligible effect on this barrier.
- Inclusion of one and two water molecules to describe explicitly the first solvation layer, within the supermolecule model, lowers the barrier considerably (29.1 and 7.6 kcal/mol).
- There is good correspondence between the DFT-predicted and experimentally reported Raman frequencies, confirming suitability of optimized geometry for the **1** as the most stable conformer of the cyanoform. This conformation is characterized also by larger HOMO–LUMO gap of 9.00 eV further confirming its marked stability.
- The NBO analysis has revealed that the lone pairs of N atoms and C–C, C–H and C–N bonds interactions give the strongest stabilization to both of the **1** and **2** with an average value of 12.5 kcal/mol.

## Additional file

10.1186/s13065-016-0166-z Selected structural parameters.
