# Supplementary material for: Tautomerization, acidity, basicity, and stability of cyanoform: a computational study
Source: Chem Cent J. 2016 Apr 11;10:20. doi: 10.1186/s13065-016-0166-z (PMC4828767; doi:10.1186/s13065-016-0166-z)
Supplement: Supplementary file 1 — 10.1186/s13065-016-0166-z Selected structural parameters. [file 13065_2016_166_MOESM1_ESM.docx]

Table 1S: Selected structural parameters for the tautomers of the cyanoform calculated in the gas phase and in the solution. .

|  |  | **gas** | | | **solution** | | |
| --- | --- | --- | --- | --- | --- | --- | --- |
|  | **bonds** | **1** | **2** | **TS** | **1** | **2** | **TS** |
| **B3LYP** | **C-H** | 1.099 |  | 1.945 | 1.110 |  | 1.867 |
|  | **C-C** | 1.474 | 1.426  1.339 | 1.411  1.430 | 1.475 | 1.422  1.188 | 1.411  1.428 |
|  | **C-N** | 1.150 | 1.155  1.198 | 1.158  1.205 | 1.150 | 1.156  1.188 | 1.197  1.158 |
|  | **N-H** | …. | 1.018 | 1.714 | ….. | 1.018 | 1.764 |
| **MP2** | **C-H** | 1.099 |  | 1.862 |  | | |
|  | **C-C** | 1.475 | 1.430  1.342 | 1.430  1.384 |  |  |  |
|  | **C-N** | 1.175 | 1.178  1.214 | 1.177  1.239 |  |  |  |
|  | **N-H** |  | 1.019 | 1.539 |  |  |  |
| **ωB97XD** | **C-H** | 1.098 |  | 1.802 |  |  |  |
|  | **C-C** | 1.472 | 1.423  1.335 | 1.417  1.416  1.412 |  |  |  |
|  | **C-N** | 1.147 | 1.151  1.195 | 1.153  1.190 |  |  |  |
|  | **N-H** | 1.015 | 1.015 | 1.661 |  |  |  |

Table 2S: The atomic natural charges of the **1**, **2**, and TS using B3LYP/6-311++G** level of theory.


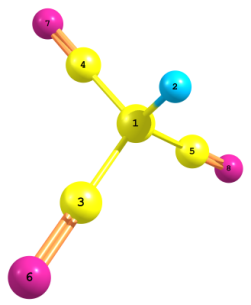


| **Atoms** | **1** | **2** | **TS** |
| --- | --- | --- | --- |
| **C1** | -0.480 | -0.444 | -0.416 |
| **H2** | 0.215 | 0.408 | 0.495 |
| **C3** | 0.289 | 0.284 | 0.266 |
| **C4** | 0.289 | 0.284 | 0.266 |
| **C5** | 0.289 | 0.538 | 0.110 |
| **N6** | -0.234 | -0.277 | -0.262 |
| **N7** | 0.234 | -0.277 | -0.262 |
| **N8** | -0.234 | -0.516 | -0.202 |


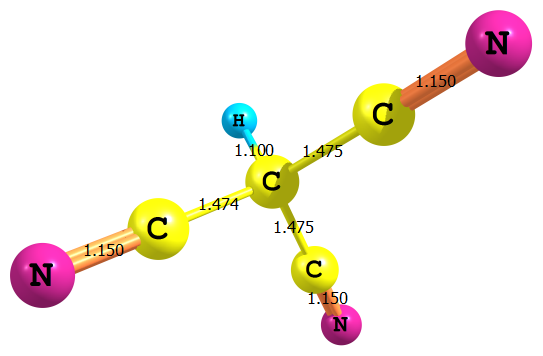


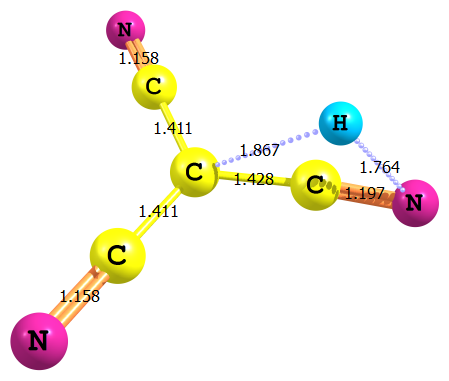


**1**

**TS**


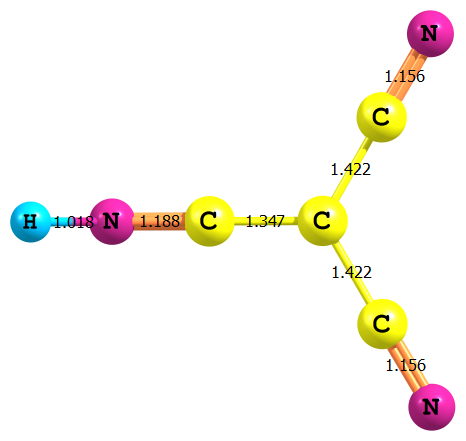

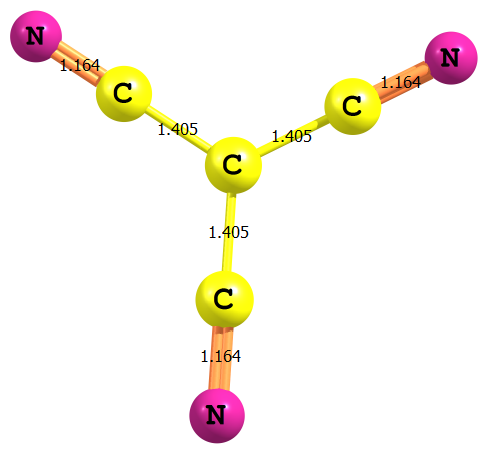


**CF^-^ 2**

**Figure 1S: Optimized structures of 1 , TS, 2 and CF^-^ obtained at the B3LYP/6-311++G** level in solution using PCM method. Bond length is in Angstrom.**
